# Preventing Growth Stagnation and Premature LH Surge Are the Keys to Obtaining a Viable Embryo in Monofollicular IVF Cycles: A Retrospective Cohort Study

**DOI:** 10.3390/jcm11237140

**Published:** 2022-12-01

**Authors:** Xiaoyan Guo, Xiaoping Zhu, Xiangli Wu, Yiqi Yu, Ling Zhang, Jing Shu

**Affiliations:** Center for Reproductive Medicine, Department of Reproductive Endocrinology, Zhejiang Provincial People’s Hospital, Affiliated People’s Hospital, Hangzhou Medical College, Hangzhou 310014, China

**Keywords:** monofollicular IVF cycles, ovarian stimulation, dual stimulation, PPOS, natural cycle, antagonist, premature LH surge, growth stagnation

## Abstract

How LH levels influenced the outcomes of monofollicular IVF cycles using different stimulation protocols was controversial. In this single-center, retrospective study, we analyzed 815 monofollicular IVF cycles between 2016–2022 using natural cycle (NC), medroxyprogesterone acetate (MPA) or clomiphene citrate (CC) in addition to human menopausal gonadotropin (hMG), with or without GnRH antagonist. A viable embryo was obtained in 35.7% of all cycles. Growth stagnation and premature LH surge are two markedly negative factors for obtaining viable embryos (odds ratios of 0.12 [0.08–0.65], *p* < 0.0001 and 0.33 [0.26,0.42], *p* < 0.0001, respectively). NC/hMG cycles are prone to premature LH surge (40.4%), yielding a significantly lower opportunity of obtaining embryos (24.7%, *p* = 0.029). The administration of GnRH antagonist on the background of MPA resulted in a significant decrease in LH levels (from 2.26 IU/L to −0.89 IU/L relative to baseline, *p* = 0.000214), leading to a higher risk of growth stagnation (18.6%, *p* = 0.007). We hypothesized that the abrupt decline of LH might increase the risk of apoptosis in granulosa cells. We proposed a “marginal effect” framework to emphasize that the change of LH was the key to its bioactivity, rather than the traditional “window” concept with fixed cutoff values of a threshold and a ceiling.

## 1. Introduction

According to the POSEIDON (Patient-Oriented Strategies Encompassing IndividualizeD Oocyte Number) classification, patients with poor ovarian reserve (POR) (antral follicle count < 5 and anti-Müllerian hormone (AMH) < 1.2 ng/mL) face a poor prognosis during in vitro fertilization (IVF) cycles [1,2]. Among them, those with a single dominant follicle are of particular difficulty since both the quality and quantity of the oocytes are reduced [3]. Monofollicular IVF cycles were reported to have an oocyte retrieval rate of about 80% [4,5] and a fertilization rate of 63.9%, while the viable embryo rate was as low as 28–62.7%, the implantation rate was 9.5–15% and the live birth rate was 2.6–8.4% per stimulation cycle [6,7,8]. 

The debate on how to improve their prognosis has intensified. Some adopted a “back to nature” attitude, advocating the natural cycle (NC) in the hope of saving money, reducing hormonal treatment and avoiding cryopreservation of the embryo. However, 16.6% of NC IVF cycles ended up with premature ovulation or untimely luteinizing hormone (LH) surges with individualized schedules for oocyte retrieval [9]. Moreover, data showed that natural cycle IVF in Bologna POR patients had a live birth rate of only 2.6% per cycle and only 7% after six NC IVF cycles cumulatively [8]. Some have proposed adding a gonadotrophin-releasing hormone antagonist (GnRHant) at the mid-late follicular phase in order to prevent LH surge. Falling short of expectations, modified NC failed to show an advantage [10,11,12]. Some studies reported that dual stimulation with clomiphene citrate and human menopausal gonadotropin (CC + hMG) yielded significantly more viable embryos than conventional antagonist protocol [13,14], offering another solution. More recently, the PPOS (progestin-primed ovarian stimulation) protocol using medroxyprogesterone acetate (MPA) has been used to effectively prevent LH rise in poor responders [15,16,17]. With a significantly lower incidence of LH surge, PPOS cycles yielded more embryos and a higher cumulative live birth rate than the flexible antagonist protocol [18], despite a larger amount of total gonadotropin use and a longer duration of stimulation [19]. 

We hypothesized that the profile of LH in various protocols would profoundly influence the quality of follicles. In this study, we aim to (1) compare the efficiency of obtaining a viable embryo in a monofollicular IVF cycle using different stimulation protocols, (2) track cycle characteristics of different stimulation protocols, especially the trend of LH, and (3) evaluate the effect of a premature LH surge and growth stagnation secondary to low LH.

## 2. Materials and Methods

### 2.1. Study Design, Patient Selection and Eligibility Criteria

In this single-center, retrospective study, we analyzed monofollicular cycles in POR patients between 2016–2022. Stimulation protocols were grouped by two factors, (1) drugs that manipulate negative feedback (levels: MPA/CC/NC), (2) GnRH antagonist usage (levels: with or without GnRH antagonist). The criterion for the dominant follicle was a diameter reaching or over 12 mm on the day of oocyte retrieval. The exclusion criteria were as follows: (1) ovarian stimulation protocols other than the aforementioned ones, such as the GnRH agonist protocol and stimulation with letrozole, (2) patients with known genetic abnormalities, such as mosaic Turner syndrome, FSHR and FOXL2 mutations [20], (3) AMH greater than 1.1 ng/mL with monofollicle development due to poor response, (4) cycles cancelled due to social reasons. The study was approved by the ethics committee of the Zhejiang Provincial People’s Hospital (QT2022395).

### 2.2. Ovarian Stimulation and Oocyte Retrieval

Follicular monitoring by transvaginal ultrasound and serum testing of follicle stimulating hormone (FSH), luteinizing hormone (LH), estrogen (E2) and progesterone (P) started on menstrual cycle day 2. The dual stimulation group (CC + hMG) used CC (clomiphene citrate, 50–100 mg/d, Codal Synto Ltd., Limassol, Cyprus) and hMG (human menopausal gonadotropin, Livzon Pharma, Zhuhai, China). The PPOS group used MPA (medroxyprogesterone acetate, 4–6 mg/d, Xianju Pharma, Taizhou, China) and hMG. The dosage of hMG ranged from 75 IU/d to 225 IU/d depending on body weight and previous stimulation history. The indication for GnRH antagonist administration (ganirelix, 0.0625–0.25 mg/day, Merck Sharpg & Dohme Limited, Hertfordshire, United Kingdom) for PPOS and dual stimulation groups was the early rise of LH surge. The indication for GnRH antagonist administration for the NC/hMG + GnRHant group was the diameter of the follicle reaching 14 mm. Serum LH levels were monitored daily when the diameter of the follicle reached 14 mm. Triggering by triptorelin (0.1 mg, Decapeptyl, Ferring Pharma, Saint-Prex, Switzerland) and ovidrell (250 mg, Merck Serono, Modugno, Italy) was given when the follicles reached maturation in terms of diameter and estrogen levels, or when an irreversible premature LH surge occurred. Transvaginal oocyte retrieval was scheduled 36 h after triggering or brought forward in case of a premature LH surge.

### 2.3. Insemination and Embryo Culture

Fertilization of the oocytes was performed by IVF or ICSI, depending on semen parameters and previous fertilization history. Zygotes were cultured in Sydney IVF cleavage medium (Cook Medical, Brisbane, Australia). Embryos were scored by the cell number, symmetry and fragmentation rate of the blastomere on the third day, according to Istanbul’s criteria [21]. Grade A and Grade B embryos were cryopreserved or transferred, while Grade C embryos underwent further blastocyst culture in Sydney IVF Blastocyst medium (Cook Medical, Brisbane, Australia) under routine conditions. Blastocysts of good or fair morphology were frozen on day 5 or day 6. 

### 2.4. Endometrial Preparation, Embryo Transfer and Pregnancy Outcome

Endometrial preparation was either by hormonal replacement (estradiol valerate tablets, 3 mg two times per day, Bayer Pharma, Leverkusen, Germany) or natural cycle. After twelve to fourteen days, dydrogesterone (10 mg two times per day, Abbott Pharma, Chicago, IL, USA), soft vaginal progesterone capsules (200 mg once per day, Cyndea Pharma, Olvega, Spain) and progesterone oil injections (40 mg once per day, Xianju Pharma, Taizhou, China) were administered. If pregnancy was achieved, the luteal support would be continued until 10 weeks of gestation. We excluded double embryo transfers with one embryo from the monofollicle cycles and the other from other conditions, resulting in 119 single embryo transfer cycles. Some embryos were still waiting to be transferred. Clinical pregnancy was defined as the presence of a gestational sac with embryonic heart activity under ultrasound examination at 7 weeks of pregnancy. Early miscarriage was defined as the spontaneous termination of pregnancy before 12 weeks’ gestation. The live birth rate was defined as the proportion of patients with live births among all transfer cycles. There was no late miscarriage, intrauterine fetal demise or multiple pregnancies.

### 2.5. Statistical Analysis

The primary outcome was whether a viable embryo was obtained in the monofollicular cycle. Secondary outcomes included the incidence of premature LH surge, the incidence of growth stagnation, the cancellation of oocyte retrieval due to premature ovulation, emergency oocyte retrieval, the number of mature oocytes, the fertilization rate, the cleavage rate, and pregnancy outcomes after embryo transfer. Growth stagnation is defined as (1) E2 declining over 50 ng/mL after the use of a GnRH antagonist, (2) E2 decreasing without using a GnRH antagonist, or (3) the growth of E2 plateauing twice or for three consecutive days. LH surge is defined as (1) LH levels over 10 IU/L or a 2-fold increase above the baseline, and (2) an apparent elevation of progesterone, despite the fact that LH elevation might not be captured.

For continuous variables, normality was tested by the Kolmogorov–Smirnov test. Continuous data were presented as mean ± standard deviation if normally distributed. Otherwise, they were shown as the median (first quartile–third quartile). Continuous variables were compared using one-way ANOVA or Kruskal–Wallis tests. The repeated measurements of LH levels at different time points were compared by the Friedman test, followed by the pairwise Wilcoxon signed-rank test for subgroup comparisons. Categorical variables were compared using a chi-squared test or Fisher exact test. Bonferroni correction was used to adjust *p*-values for multiple testing [22]. *p* < 0.05 was considered statistically significant. Binary logistic regression was used to investigate the independent effects of growth stagnation and premature LH surge on the odds of obtaining a viable embryo, with the adjustment for stimulation protocol, AMH, baseline FSH and LH. The adjusted odds ratio (OR) with a 95% confidence interval was calculated by the coefficient of the variable in the regression model. All data were analyzed and plotted with R (4.2.0).

## 3. Results

### 3.1. Patient and Cycle Characteristics

We included 815 monofollicular IVF cycles from 417 POR patients, with an average age of 38.6 ± 7.3 years and AMH of 0.44 ± 0.41 ng/mL (Table 1). Basic characteristics of the patients are shown in Table 1. Age, BMI, the proportion of primary infertility, infertility duration, the number of previous IVF failures and infertility causes were comparable among all groups. However, AMH and baseline hormonal levels are significantly different among these groups, with the NC/hMG group having significantly lower AMH and higher baseline FSH and LH levels than the CC + hMG + GnRHant group. These characteristics are therefore included as confounding factors in the regression equation.

### 3.2. IVF Outcome

Among the 815 monofollicular cycles, 5.3% were cancelled due to premature ovulation and 31.8% failed to obtain a normal oocyte (Table 2). A total of 62.9% of the cycles yielded a normal MII oocyte, and 47.1% of the cycles yielded a 2PN fertilized zygote. Finally, a viable embryo was obtained in only 35.7% of the cycles. Across all regimens, NC/hMG had the lowest opportunity for achieving a viable embryo, whereas MPA + hMG had a significantly higher chance (24.7% vs. 41.7%, *p* = 0.03). 

Premature LH surge emerged in 16.7% of cycles, especially in NC/hMG cycles, and 7.8% ended up with premature ovulation and 24.7% without emergency oocyte retrieval. Premature LH surge is the primary drag on the chance of obtaining an embryo in NC/hMG cycles. Timely administration of antagonist in the NC/hMG + GnRHant group lowers the risk of premature LH surge by half (40.4% vs. 20%, *p* = 0.0075), leading to a higher chance of achieving a mature oocyte (from 51.8% to 66.9%) and a viable embryo (from 24.7% to 38.1%). 

Growth stagnation had an overall incidence of 10.2%. MPA + hMG group was susceptible to growth stagnation (10.8%), especially when antagonist was added (18.6%), which was significantly higher than the NC/hMG group (4.8%, *p* = 0.0075). Owing to the inhibitory effect of MPA, the risk of premature LH surge was significantly lower in the MPA + hMG group than the NC/hMG group (8.8% vs. 40.4%, *p* = 0.0075).

### 3.3. LH Profile and the Effect of Growth Stagnation and LH Surge

**Figure 1:**
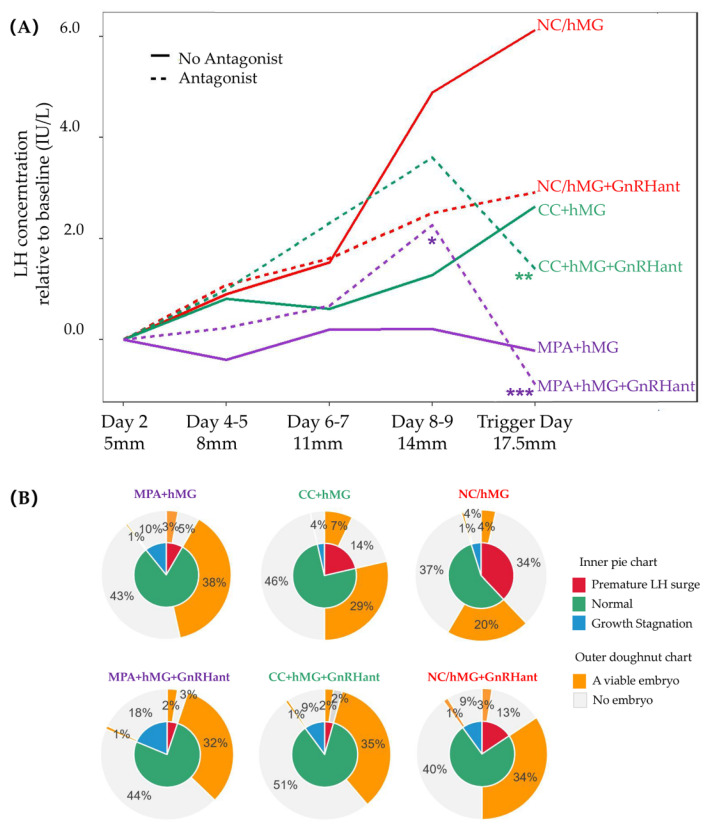
LH profile in monofollicular IVF cycles using various stimulation protocols. (**A**) LH levels were shown as the difference relative to baseline. * denoted that LH levels at this time point were significantly different from the previous measurement, *: *p* < 0.05, **: *p* < 0.01, ***: *p* < 0.001. The means, standard deviations and *p* values of pairwise comparisons are listed in Supplementary Table S2 out of aesthetic considerations. (**B**) The inner pie chart denotes the proportions of growth stagnation (blue), premature LH surge (red) and normal growth (green) in monofollicular IVF cycles. The outer doughnut chart showed the chances of obtaining a viable embryo (golden) or not (grey) on the conditions of growth stagnation, LH surge and normal growth, respectively.

Serum LH levels in various stimulation protocols are presented in Figure 1A, shown as the relative difference to baseline. In NC/hMG cycles, LH levels continued to rise in the process of follicular development, with two fifths of cycles experiencing a premature LH surge (Figure 1B). Compared with NC/hMG cycles, NC/hMG with GnRH antagonist administration offered better control of LH levels (Figure 1A, red dashed line) and significantly reduced the risk of LH surge, leading to a higher chance of obtaining a viable embryo (from 24.7% to 38.1%).

For PPOS cycles, some might experience an early rise in LH levels (Figure 1A, purple dashed line). The administration of GnRH antagonists on the background of MPA resulted in a sharp decrease in LH levels (from 2.26 IU/L to −0.89 IU/L relative to baseline, *p* = 0.000214). The abrupt drop in LH led to a higher risk of growth stagnation (from 10.8% to 18.6%). The pattern held true for dual stimulation cycles. On the occasion of a commencing LH rise in CC + hMG cycles, GnRH antagonists helped to prevent LH surge (Figure 1A, green dashed line). However, after the administration of GnRH antagonists in addition to CC, the serum LH levels significantly dropped 2.2 IU/L (*p* = 0.002), leading to a higher risk of growth stagnation (from 3.6% to 10.1%). 

Once growth stagnation occurred, the chances of obtaining an embryo dropped to as low as 8.3%. This pattern was consistent across all protocols, as shown in Figure 1B. The odds ratio of obtaining an embryo after growth stagnation is 0.12 [0.08–0.65], *p* < 0.0001, after the adjustment of stimulation protocol, AMH, baseline FSH and LH. LH surge also greatly dampened the hope of getting an embryo (14.7%), with an OR of 0.33 [0.26,0.42], *p* < 0.0001 after adjustment. 

### 3.4. Pregnancy Outcomes of Embryo Transfer Cycles

We tracked the pregnancy outcomes of embryos originating from monofollicular cycles (Table 3). A total of 119 single embryo transfer cycles were completed at the time of manuscript submission. Endometrial thickness, the proportion of frozen embryo transfers, endometrial preparation protocol and the type of embryos being transferred were comparable among all groups. The overall clinical pregnancy rate per transfer was 24.4%. Surprisingly, embryos from the natural cycle (NC/hMG) and modified natural cycle (NC/hMG + GnRHant) yielded the highest clinical pregnancy rates (38.1% and 36.7%) and live birth rates (23.8% and 13.3%), although they did not reach statistical significance due to the limited sample size.

## 4. Discussion

To the best of our knowledge, our study provided the first ever evidence of how LH levels in monofollicular IVF cycles using different protocols affected clinical outcomes. As for the choice of stimulation protocol, MPA + hMG had the highest chance of achieving a viable embryo, while NC/hMG had a significantly lower chance. After further analysis, we found that the main difference among various regimens lay in the control of LH. NC had no restriction on LH, while MPA, CC and GnRH antagonists exerted different effects of LH inhibition. Preventing growth stagnation and premature LH surges are the keys to obtaining a viable embryo in monofollicular IVF cycles. 

It is undisputed that there is a “window” for LH to enable normal follicle and oocyte development [23]. LH levels below the “threshold” would inhibit the function of granulosa cells, leading to slow response and growth stagnation. LH levels above the “ceiling” would lead to improper resumption of meiosis, luteinization of granulosa cells and premature ovulation [24]. However, the actual upper and lower limits of LH necessary for proper folliculogenesis presented high inter-individual and inter-cycle heterogeneity. According to our study, apart from the widely acknowledged importance of absolute LH levels, the impact of its intra-cycle change was underestimated. As are shown in Figure 1, the administration of GnRH antagonist on the background of MPA resulted in a significant decrease in LH levels, leading to a higher risk of growth stagnation and ending up with a lower chance of achieving a viable embryo. It is worth noting that even after the sudden drop in LH after GnRH antagonist administration, the absolute concentrations of LH were still comparable to baseline. We hypothesized that an abrupt decline of LH might increase the risk of apoptosis of granulosa cells, leading to growth stagnation of the follicle and unsatisfactory outcomes. We proposed a “marginal effect” framework to emphasize the change of LH rather than the traditional “window” concept with fixed cutoff values of a threshold and a ceiling.

For patients with poor ovarian reserve, they face a tricky situation where they have the risk of a premature LH surge on the one hand and the risk of growth stagnation secondary to relative LH deficiency on the other hand. We found that these were the two major negative factors for obtaining viable embryos (odds ratios of 0.12 [0.08–0.65], *p* < 0.0001 and 0.33 [0.26,0.42], *p* < 0.0001, respectively). Premature LH surge was most commonly seen in the NC/hMG group, which was characterized by no external suppression on LH levels. Growth stagnation was most commonly seen in the MPA + hMG + GnRHant group, which was characterized by tough suppression of LH levels. In fact, if we were able to keep LH levels at an optimal level for POR patients, as in the MPA + hMG group, we were more likely to have a viable embryo (41.7%).

The preovulatory stage is a critical phase where granulosa cells are receptive to and dependent on LH to sustain follicular growth [16]. After LH binds to its receptor LHCGR in granulosa cells, it simultaneously induces the activation of multiple intracellular signaling cascades, including the classical cAMP/PKA (Cyclic adenosine 3′,5′-monophosphate/protein kinase A) pathway [25], resulting in the phosphorylation of ERK1/2 (extracellular-regulated kinase) and CREB (cAMP-responsive element binding protein) and the transcription of steroidogenic genes, such as StAR (steroidogenic acute regulatory protein), P450scc (cytochrome P450 side chain cleavage) and aromatase, leading to an elevation of estrogen synthesis [26,27,28]. Apart from the steroidogenic effect, LH also promotes granulosa cell proliferation [29] and mediates anti-apoptotic effects [30] via the PI3K/AKT (phosphatidylinositol 3-kinases/protein kinase B) pathway by the activation of STARD1, CCND2 and XIAP gene expression [31]. It is not surprising that reduced LH activities would lead to apoptosis of ovarian granulosa cells [32].

Different medications, such as MPA, CC and GnRH antagonists, exert specialized effects on LH levels. MPA was proven to slow LH pulse frequency, lower pulse amplitude and reduce plasma LH levels [33]. However, this effect of LH surge prevention would fail if MPA administration was started after follicles became dominant and serum estrogen levels were already elevated [34]. Research has found that administration of progesterone inhibits murine granular cell proliferation and reduces the growth rate of follicles via the PI3K/AKT and MAPK pathways [35]. Our study also found that follicle growth was especially vulnerable to stagnation if a GnRH antagonist was used along with MPA.

Clomiphene contains a mixture of two isomers, about 2/3 in the enclomifene (trans) form and 1/3 in the zuclomifene (cis) form [36]. Clomiphene’s primary mechanism of action comes from the estrogen receptor antagonist effects of enclomifene in the hypothalamus, where it enhances the release of GnRH, resulting in FSH and LH secretion by the pituitary that, in turn, stimulates follicular growth. The anti-estrogenic effect of enclomiphene might not only block the negative feedback of estrogen but also its positive feedback, resulting in the prevention of the LH surge [37]. Therefore, CC first provided a stimulatory effect through the negative feedback and then a weak inhibitory effect. As enclomifene is eliminated rapidly within 24 h, clomiphene tablets need to be given once a day until the day of ovulation trigger in order to prevent LH surge. In contrast, zuclomifene probably has estrogen agonist actions at the pituitary level, with a half-life of about 5 days [38]. Clinical studies also supported the effect of LH surge prevention on CC after continuous use until triggering. CC reduced the rate of premature ovulation from 27.8% in the natural cycle group to 6.8% with CC (25 mg/day, from day 7 to triggering) (*p* < 0.001) in IVF patients, with half of the participants suffering from poor ovarian reserve [39]. In another study of patients receiving intrauterine insemination due to mild male factor or unexplained infertility, the rate of premature LH surge was also significantly lower in the hMG + CC group (CC 50 mg tid, from day 4 to triggering) than the hMG group (5.45% vs. 15.89%) [40]. Our data also supported that continuous use of CC reduced the rate of premature LH surge from 40.4% to 21.4%. However, the effect of CC on LH surge prevention was not as reliable or rapid as GnRH antagonist. Sometimes, a premature LH release might override the anti-estrogenic effect, and the administration of GnRH antagonist in addition to CC was able to further reduce the risk of premature LH surge to 5% in monofollicular cycles.

What is the best strategy to deal with the beginning of a LH surge? One choice is to trigger immediately, with some successful experience being reported [41,42]. Another choice is to use GnRH antagonists to block the rise. In clinical practice, we would take the diameter of the follicle, the progesterone levels and the type of regimen into consideration. According to our study, adding GnRH antagonists in cycles with MPA and CC posed the risk of growth stagnation. Theoretically, the addback of recombinant LH in addition to the GnRH antagonist might alleviate the risk, but more evidence would be needed.

There are several limitations to this study. Firstly, the retrospective nature led to heterogeneous baseline characteristics among different regimen groups. The NC/hMG group had significantly lower AMH and higher baseline FSH and LH, which were important confounders of the outcome. The lack of randomization weakened the power of the evidence. Secondly, the decision of whether GnRH antagonist would be used was made according to the actual clinical situation rather than planned beforehand, so the two subgroups with or without GnRH antagonist are inherently different in terms of the day 8 situation, which might interfere with the outcome. Thirdly, the sample size was small, especially in the CC + hMG group. Fourth, the interval between ovulation triggering and oocyte retrieval was not analyzed in the study, but it was crucial to preventing ovulation before the retrieval while ensuring proper maturity of oocytes, especially when there was a premature LH surge [43,44]. Caution is needed when interpreting the results, and future randomized controlled trials with a larger sample size are called for.

## 5. Conclusions

This study showed that proper control of LH was the key to obtaining a viable oocyte in monofollicular IVF cycles in patients with poor ovarian reserve. Different medications, such as MPA, CC and GnRH antagonists, exert different effects on LH levels. MPA + hMG was an effective choice, but the risk of growth stagnation should be alerted. The administration of the GnRH antagonist on the background of MPA or CC might lead to a sharp decrease in LH levels, resulting in a higher risk of growth stagnation, ending up with a lower chance of achieving a viable embryo. We emphasized that the change in LH was the key to its bioactivity rather than its absolute value. 

## Figures and Tables

**Table 1:** Patient Characteristics.

	Group 1 MPA		Group 2 CC		Group 3 NC		*p* Value
	1a	1b	2a	2b	3a	3b	
	MPA + hMG	MPA + hMG + GnRHant	CC + hMG	CC + hMG + GnRHant	NC/hMG	NC/hMG + GnRHant	
	*n* = 204	*n* = 118	*n* = 28	*n* = 139	*n* = 166	*n* = 160	
Age, year	38.3 ± 6.2	39.1 ± 6.0	38.9 ± 6.2	38.7 ± 6.0	39.1 ± 6.7	37.8 ± 6.9	0.430
BMI, *kg/m2*	21.9 ± 2.8	21.9 ± 2.8	22.3 ± 2.6	22.4 ± 2.8	22.2 ± 3.0	21.8 ± 2.7	0.461
AMH, *ng/mL*	0.39 ± 0.33	0.42 ± 0.35	0.65 ± 0.48 *^,^^	0.54 ± 0.41 *^,^^	0.38 ± 0.34	0.47 ± 0.45	<0.001
Primary Infertility, *n (%)*	69 (33.8%)	36 (30.5%)	9 (32.1%)	48 (34.5%)	51 (30.7%)	54 (33.8%)	0.967
Infertility duration, year	4.1 ± 3.8	4.8 ± 4.3	4.2 ± 3.3	4.0 ± 3.7	4.5 ± 4.5	4.1 ± 3.7	0.595
Previous IVF failures, *n*	2.9 ± 3.3	2.9 ± 3.7	2.6 ± 2.9	2.5 ± 3.3	3.0 ± 3.1	2.8 ± 3.0	0.788
Infertility causes							
Tubal factor, *n (%)*	87 (42.6%)	57 (48.3%)	9 (32.1%)	53 (38.1%)	67 (40.4%)	61 (38.1%)	0.132
Endometriosis, *n (%)*	25 (12.3%)	14 (11.9%)	3 (10.7%)	17 (12.2%)	24 (14.5%)	29 (18.1%)	0.086
Male factor, *n (%)*	142 (69.6%)	89 (75.4%)	18 (64.3%)	101 (72.7%)	108 (65.1%)	113 (70.6%)	0.116
Unexplained, *n (%)*	26 (12.7%)	17 (14.4%)	8 (28.6%)	18 (12.9%)	25 (15.1%)	20 (12.5%)	0.152
Day 2 FSH, *IU/L*	12.3(8.6)	13.5 (7.3)	8.4 (4.5)	9.0 (5.0) *^,#,^^	13.5 (9.4)	10.5 (5.1)	<0.001
Day 2 LH, *IU/L*	4.5 (4.3)	5.1 (3.4)	3.2 (1.4)	3.7 (2.4)^	6.1 (4.5)	4.9 (4.5)	0.003
hMG dose, *IU*	856.62 ^ (487.82)	959.04 ^ (404.69)	668.06 (609.30)	824.32 ^ (620.97)	507.95 (484.92)	725.94 (567.52)	<0.001
hMG duration, day	7.61 (2.48)	8.17 (2.06)	5.52(3.64) *^,$^	6.61(2.80) *^,$,^^	4.99(3.46) *^,$^	6.05 (2.96) *^,$^	<0.001

Notes: MPA: medroxyprogesterone acetate, hMG: human menopausal gonadotropin, GnRHant: GnRH antagonist (Ganirelix), CC: clomiphene citrate, NC: natural cycle. Bonferroni correction was used to adjust *p*-values for multiple testing. *p* Values of pairwise comparisons are listed in Supplementary Table S1. *: significantly different from MPA + hMG group; ^#^: significantly different from MPA + hMG + GnRHant group; ^: significantly different from NC/hMG group; ^$^: significantly different from NC/hMG + GnRHant group.

**Table 2:** Cycle characteristics and outcomes of oocytes and embryos.

	Group 1 MPA		Group 2 CC		Group 3 NC		*p* Value
	1a	1b	2a	2b	3a	3b	
	MPA + hMG	MPA + hMG + GnRHant	CC + hMG	CC + hMG + GnRHant	NC/hMG	NC/hMG + GnRHant	
	*n* = 204	*n* = 118	*n* = 28	*n* = 139	*n* = 166	*n* = 160	
Premature LH surge, *n* (%)	18 (8.8%) ^^,$^	6 (5.1%) ^^,$^	6 (21.4%)	7 (5.0%) ^^,$^	67 (40.4%)	32 (20%) ^	<0.001
Growth stagnation, *n* (%)	22 (10.8%)	22 (18.6%) ^	1 (3.6%)	14 (10.1%)	8 (4.8%)	16 (10.0%)	0.007
Premature ovulation, *n* (%)	11 (5.4%) ^	6 (5.1%) ^	0 (0%)	4 (2.9%) ^	13 (7.8%)	9 (5.6%) ^	0.371
Emergency retrieval, *n* (%)	16 (7.8)	5 (4.2%)	2 (7.1%)	7 (5.0%)	41 (24.7%)	19 (11.9%)	<0.001
Oocytes retrieved, *n* (%)	153 (75.0%) ^	73 (61.9%)	20 (71.4%)	109 (78.4%) ^^,#^	99 (59.6%)	119 (74.4%) ^	0.001
Abnormal	14 (6.9%)	6 (5.1%)	2 (7.1%)	8 (5.8%)	10 (6.0%)	9 (5.6%)	0.99
GV/MI	1 (0.5%)	1 (0.8%)	0 (0%)	3 (2.2%)	3 (1.8%)	3 (1.9%)	0.698
MII	138 (67.6%) ^	66 (55.9%)	18 (64.3%)	98 (70.5%) ^	86 (51.8%)	107 (66.9%)	0.003
ICSI, *n* (%)	47 (33%)	25 (37.3%)	8 (42.1%)	55 (39.6%)	47 (28.3%)	34 (29.6%)	0.145
2PN zygotes, *n* (%)	103 (53.4%)	53 (47.7%)	14 (50.0%)	74 (54.8%)	60 (39.5%)	80 (53.0%)	0.237
Cleaved embryos, *n* (%)	97 (50.8%)	50 (45.0%)	13 (46.4%)	72 (53.3%) ^	53 (35.1%)	76 (50.3%)	0.026
Top quality embryos, *n* (%)	74 (36.3%)	29 (24.6%)	8 (28.6%)	39 (28.1%)	25 (15.2%)	46 (28.9%)	0.001
Viable embryos, *n* (%)	85 (41.7%) ^	42 (35.6%)	10 (35.7%)	52 (37.4%)	41 (24.7%)	61 (38.1%)	0.029
Day 3 embryos	76 (37.3%) ^	34 (28.8%)	9 (32.1%)	43 (30.9%)	36 (21.7%)	55 (34.4%)	0.041
Day 5/6 embryos	9 (4.4%)	8 (6.8%)	1 (3.6%)	9 (6.5%)	5 (3.0%)	6 (3.8%)	0.603

Notes: All proportions are calculated among the total number of initiated stimulation cycles. MPA: medroxyprogesterone acetate, hMG: human menopausal gonadotropin, GnRHant: GnRH antagonist (Ganirelix), CC: clomiphene citrate, NC: natural cycle. Bonferroni correction was used to adjust *p*-values for multiple testing. *p* Values of pairwise comparisons are listed in Supplementary Table S1. ^#^: significantly different from MPA + hMG + GnRHant group; ^: significantly different from NC/hMG group; ^$^: significantly different from NC/hMG + GnRHant group.

**Table 3:** Pregnancy outcomes of embryos originated from the cycles.

	Group 1 MPA		Group 2 CC		Group 3 NC		*p* Value
	1a	1b	2a	2b	1a	1b	
	MPA + hMG	MPA + hMG + GnRHant	CC + hMG	CC + hMG + GnRHant	NC/hMG	NC/hMG + GnRHant	
	*n* = 33	*n* = 14	*n* = 6	*n* = 15	*n* = 21	*n* = 30	
Endometrial thickness, mm	9.20 ± 2.10	9.36 ± 2.03	9.12 ± 1.78	11.17 ± 2.50	9.47 ± 2.31	9.42 ± 2.50	0.124
Frozen embryo transfer, *n* (%)	33 (100%)	14 (100%)	6 (100%)	14 (93.3%)	19 (90.5%)	27 (90%)	0.671
Endometrial preparation by hormone replacement, *n* (%)	29 (87.9%)	13 (92.9%)	6 (100%)	14 (93.3%)	18 (85.7%)	26 (86.7%)	0.890
Day 3 embryos, *n* (%)	31 (93.9%)	13 (92.9%)	5 (83.3%)	11 (73.3%)	19 (90.5%)	28 (93.3%)	0.311
Top quality embryos, *n* (%)	30 (90.9%)	8 (57.1%)	5 (83.3%)	11 (73.3%)	13 (65.0%)	22 (73.3%)	0.142
Clinical pregnancy, *n* (%)	6 (18.2%)	1 (7.1%)	0 (0.0%)	3 (20.0%)	8 (38.1%)	11 (36.7%)	0.086
Biochemical pregnancy, *n* (%)	3 (9.1%)	1 (7.1%)	3 (50.0%)	0 (0.0%)	3 (14.3%)	3 (10.0%)	0.082
Miscarriage, *n* (%)	1 (3.0%)	1 (7.1%)	0 (0.0%)	1 (6.7%)	2 (9.5%)	6 (20.0%)	0.338
Ectopic pregnancy, *n* (%)	1 (3.0%)	0 (0.0%)	0 (0.0%)	0 (0.0%)	0 (0.0%)	1 (3.3%)	1.000
Ongoing pregnancy, *n* (%)	1 (3.0%)	0 (0.0%)	0 (0.0%)	0 (0.0%)	1 (4.8%)	0 (0.0%)	0.859
Live birth, *n* (%)	3 (9.1%)	0 (0.0%)	0 (0.0%)	2 (13.4%)	5 (23.8%)	4 (13.3%)	0.393

Notes: Some embryos were still waiting to be transferred. MPA: medroxyprogesterone acetate, hMG: human menopausal gonadotropin, GnRHant: GnRH antagonist (Ganirelix), CC: clomiphene citrate, NC: natural cycle.

## Data Availability

Data would be available on request from the corresponding author.

## Supplementary Materials

The following supporting information can be downloaded at: https://www.mdpi.com/article/10.3390/jcm11237140/s1, Table S1: Adjusted *p* values for pairwise comparisons; Table S2: Detailed data of Figure 1A.
